# Reviewer acknowledgement 2015

**DOI:** 10.1186/s40409-016-0060-0

**Published:** 2016-02-04

**Authors:** Benedito Barraviera

**Affiliations:** Journal of Venomous Animals and Toxins including Tropical Diseases, Center for the Study of Venoms and Venomous Animals (CEVAP), São Paulo State University (UNESP – Univ Estadual Paulista), Botucatu, SP Brazil

## Abstract

The Editor of *Journal of Venomous Animals and Toxins including Tropical Diseases* would like to thank all of our reviewers, both external and Editorial Board Members, who have contributed to the journal in Volume 21 (2015) and whose valuable support is fundamental to the success of the journal.

**Adolfo Borges**

Venezuela

**Adolfo Rafael de Roodt**

Argentina

**Alice Martins**

Brazil

**Aline Silva**

Brazil

**Ana Roselino**

Brazil

**Ana Carolina Vasconcelos**

Brazil

**Ana Cecília Oliveira**

Brazil

**Ana Leonor Abrahão Nencioni**

Brazil

**Andre Lopes Fuly**

Brazil

**Ary Junior**

Brazil

**Benito Soto-Blanco**

Brazil

**Carla Lima**

Brazil

**Carolina Gigek**

Brazil

**Caroline Mourão**

Brazil

**Ceila Malaque**

Brazil

**Cesar Silva**

Brazil

**Chaturaka Rodrigo**

Sri Lanka

**Chester Joyner**

United States of America

**Cilmery Kurokawa**

Brazil

**Cleide Consales**

Brazil

**Daniel Horton**

United Kingdom

**Daniel Saidemberg**

Brazil

**Daniel Pimenta**

Brazil

**Elaine Fitches**

United Kingdom

**Elizabeth Rangel**

Brazil

**Emanuel Marques da Silva**

Brazil

**Emanuel Silva**

Brazil

**Eneas Carvalho**

Brazil

**Enzo Ierardi**

Italy

**Evanguedes Kalapothakis**

Brazil

**Fabiana Miraglia**

Brazil

**Fan Wen**

Brazil

**Fernanda Amorim**

Brazil

**Francielle Cordeiro**

Brazil

**Geoffrey Isbister**

Australia

**Gerardo Corzo**

Mexico

**Hiroshi Nagai**

Japan

**Ida Sano-Martins**

Brazil

**Jane Megid**

Brazil

**Jean-Philippe Chippaux**

Benin

**Jennifer Smith**

Australia

**Jose Raduan Jaber Mohamad**

Spain

**José Roberto Aparecido dos Santos Pinto**

Brazil

**José-María Gutiérrez**

Costa Rica

**Juliana Zuliani**

Brazil

**Katia Dantas**

Brazil

**Kayena Zaqueo**

Brazil

**Kelly Tudini**

Brazil

**L Chang**

Taiwan

**Lawan Chanhome**

Bangladesh

**Leonardo de Sousa**

Venezuela

**Ligia Rugolo**

Brazil

**Lucilene Delazari dos Santos**

Brazil

**Lucy Lioni**

Brazil

**Luis Alberto Ponce-Soto**

Brazil

**Luis Roberto Gonçalves**

Brazil

**Luiz Felipe Passero**

Brazil

**Luiz Fernando Izidoro**

Brazil

**Luiz Gustavo Gardinassi**

Brazil

**Marcelo Duarte**

Brazil

**Marcelo L Santoro**

Brazil

**Marcia Laurenti**

Brazil

**Maria Peichoto**

Argentina

**Maria A Mariggio**

Italy

**Maria Regina Sandoval**

Brazil

**Marjorie Golim**

Brazil

**Marta Monteiro**

Brazil

**Matthew Graham**

United States of America

**Maurício Ventura Mazzi**

Brazil

**Michael Gurevitz**

Israel

**Mirian M Mendes**

Brazil

**Najet Srairi-Abid**

Tunisia

**Narumol Pakmanee**

Thailand

**Norival Santos-Filho**

Brazil

**Palmira Cupo**

Brazil

**Patrícia Brigatte**

Brazil

**Pedro Pardal**

Brazil

**Peter Strong**

United Kingdom

**Pierre Bougis**

France

**Qiumin Lu**

China

**Renata Rodrigues**

Brazil

**Renata Costa**

Brazil

**Rick Vetter**

United States of America

**Rodrigo Soares**

Brazil

**Rosany Bochner**

Brazil

**Salvatore De-Simone**

Brazil

**Sara Luz Morales-Lázaro**

Mexico

**Sebastian Estrada**

Colombia

**Sébastien Larréché**

France

**Shin Yee Fung**

Malaysia

**Silvana Marcussi**

Brazil

**Silvio Vasconcellos**

Brazil

**Simone Lucheis**

Brazil

**Stephen Hyslop**

Brazil

**Sylvia Lucas**

Brazil

**Tatiane Paixão**

Brazil

**Thais Silveira**

Brazil

**Thiago Belinato**

Brazil

**Thomaz Rocha e Silva**

Brazil

**Valquiria Dorce**

Brazil

**Vera Petricevich**

Mexico

**Victor Fet**

United States of America

**Victor Morais**

Uruguay

**Vidal Haddad Jr**

Brazil

**Yen-Chia Chen**

Taiwan

**Zhijian Cao**

China

